# Distribution and diversity of the vectors of Rift Valley fever along the livestock movement routes in the northeastern and coastal regions of Kenya

**DOI:** 10.1186/s13071-015-0907-1

**Published:** 2015-05-28

**Authors:** Samwel O Arum, Christopher W Weldon, Benedict Orindi, Tobias Landmann, David P Tchouassi, Hippolyte D Affognon, Rosemary Sang

**Affiliations:** International Centre of Insect Physiology and Ecology, P. O. Box 30772–00100, Nairobi, Kenya; Department of Zoology and Entomology, University of Pretoria, Private Bag X20, Pretoria, Hatfield 0083 South Africa

**Keywords:** Livestock migration route (LMR), Rift Valley fever (RVF), Species richness, Vector diversity

## Abstract

**Background:**

Knowledge of vector ecology is important in understanding the transmission dynamics of vector borne disease. In this study, we determined the distribution and diversity of mosquitoes along the major nomadic livestock movement routes (LMR) in the traditional pastoral ecozone of northeastern Kenya. We focused on the vectors of Rift Valley fever virus (RVFv) with the aim of understanding their ecology and how they can potentially influence the circulation of RVFv.

**Methods:**

Mosquito surveys were conducted during the short and long rainy seasons from November 2012 to August 2014 using CO_2_-baited CDC light traps at seven sites selected for their proximity to stopover points that provide pasture, water and night bomas (where animals spend nights). We compared mosquito abundance and diversity across the sites, which were located in three ecological zones (IV, V and VI), based on the classification system of agro-ecological zones in Kenya.

**Results:**

Over 31,000 mosquitoes were trapped comprising 21 species belonging to 6 genera. Overall mosquito abundance varied significantly by ecological zones and sites. *Mansonia* species (*Ma. uniformis* and *Ma. africana*) were predominant (*n* = 12,181, 38.3 %). This was followed by the primary RVF vectors, *Ae. ochraceus and Ae. mcintoshi* comprising 17.9 and 14.98 %, respectively, of the total captures and represented across all sites and ecological zones. The Shannon diversity index ranged from 0.8 to 2.4 with significant zone, site and seasonal variations. There was also significant species richness of RVF vector across ecological zones.

**Conclusion:**

Our findings highlight differential occurrence of RVFv vectors across ecological zones and sampling sites, which may be important in determining areas at risk of emergence and circulation of RVFv. Moreover, the vector distribution map along LMR generated in this study will guide potential interventions for control of the disease, including strategic vaccination for livestock.

## Background

Rift Valley fever (RVF) is a viral disease that mainly affects livestock and humans, although many other mammalian species have been shown to be susceptible [1–3]. It causes abortions and high mortality in young animals, and in humans it presents as a non-specific flu-like syndrome through to encephalitis, and ocular or hemorrhagic syndrome [4]. RVF is caused by RVF virus (RVFv), one of the six hemorrhagic fever viruses that occur in Africa [5, 6]. Although epidemics of the disease have been occurring in sub-Saharan Africa at irregular intervals, there is limited knowledge on how the virus is maintained during inter-epidemic periods, and the factors contributing to the re-emergence of the disease in hotspot areas are poorly understood. Importantly, gaps remain in our understanding of critical aspects of the ecology of potential vectors and how the vector-virus-host interaction influences the epidemiology of RVF [3, 5, 7, 8].

RVF is a vector-borne disease usually transmitted to mammals by mosquitoes (Diptera: Culicidae), and mainly depends on the availability of competent vectors, susceptible hosts, and suitable ecological and environmental conditions that favour mosquito survival and reproduction [9, 10]. RVF vectors can be classified into two major groups, namely primary and secondary vectors. In Kenya, the known primary vectors, *Aedes mcintoshi* Huang and *Aedes ochraceus* Theobald, are believed to serve as reservoirs for the virus [9, 11, 12]. Breeding of these vectors has mostly been associated with characteristic shallow depressions on land called “dambos” [13]. The dambos are usually flooded after heavy rainfall, resulting in mass emergence of floodwater *Aedes* mosquitoes [13, 14]. The primary vectors maintain RVFv transovarially by transmitting the virus through to the eggs [13]. The infected eggs can enter diapause in dry dambos for long periods and hatch into infectious mosquitoes during periods of extended rainfall. This may result in transmission of the virus to nearby animals and human beings when the vectors seek blood meals. Once primary transmission of the virus has taken place, secondary vectors belonging to the genera *Culex*, *Anopheles* and *Mansonia*, which take over flooded grounds for breeding, contribute to the amplification of the virus due to their ubiquitous biting patterns, consequently resulting in outbreaks [4, 15–17].

Northeastern Kenya is an important hotspot for RVF in Kenya, being the region hardest hit by outbreaks in 1997/98 and 2006/07 [17, 18]. These outbreaks affected over 18 districts, herdsmen lost their lives, and large economic losses were incurred due to animal abortions and deaths, as well as a ban on livestock trade and transportation [18]. In this region, pastoralism is the main source of livelihood and income. Pastoralism is a major production strategy in which people raise herds of animals, mostly in arid and semi-arid lands. Arid and semi-arid land covers about 80 % of Kenya’s landmass, and supports about a third of the country’s human population and 70 % of the national livestock kept in large herds. Due to limited and unpredictable rains, herders practice nomadic pastoralism, moving animals in large herds in search of pasture and water. This practice also favours convergence of domestic and wild animals from time to time, which may create opportunities for cross transmission of diseases. Such an interface may serve as virus emergence points or reservoirs during the inter-epidemic period and also create variable risk points for infection of susceptible livestock.

Like most arboviruses, RVF is driven by a complex interaction of mosquito vector populations and vertebrate hosts in different habitat types under varying environmental conditions. [9, 13, 19, 20]. During previous outbreaks, key primary vectors of RVF virus were identified [20] but the limited understanding of their ecology in diverse ecological zones and the interplay with the nomadic pastoral systems along the major livestock movement routes (LMR) was unknown. For these reasons, this study sought to determine the species composition and diversity of potential RVFv vectors along a LMR in northeastern Kenya. The reported research represents part of ongoing project to track RVF prevalence in nomadic herds along LMR to identify risk foci that can be targeted for RVF prevention measures. It is also envisaged that tracking of animal movement will permit identification of areas where introduction or amplification of the disease could potentially occur from wild disease reservoirs or hosts due to a high density of RVF vector populations, which could contribute to understanding of RVF epidemiology and present opportunities for strategic disease prevention.

## Methods

### Study site

**Fig. 1:**
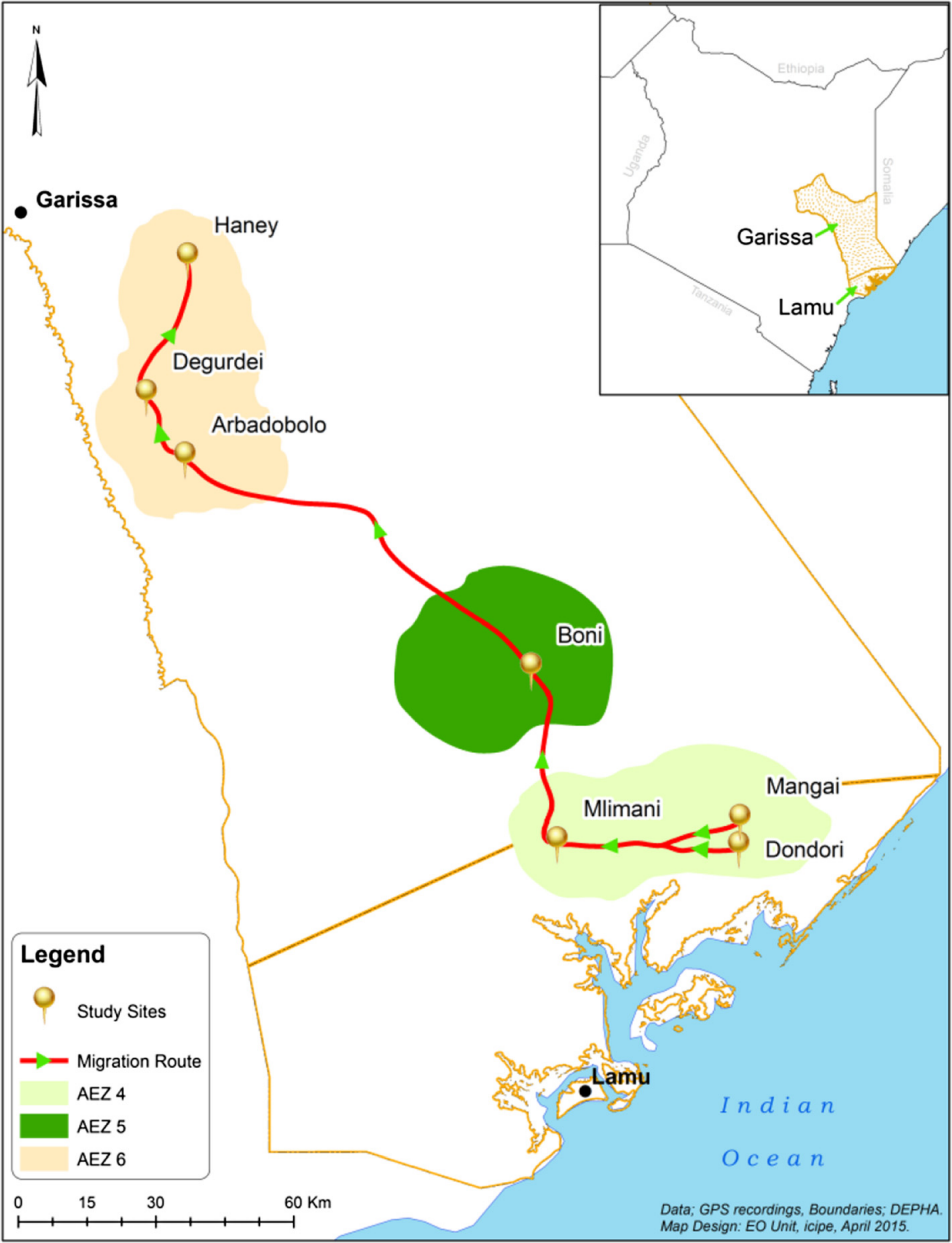
Map showing the location of study sites along the livestock movement routes in northeastern Kenya

This study was conducted along nomadic livestock movement routes (LMR) established by the tracking of a sentinel herd, which moved in search of pasture and water in northeastern province and coastal parts of Kenya (Fig. 1) stretching between Garissa S00° 39′ E40° 05′ and Lamu S2° 16′ E40° 54′ Counties. Garissa County is traditionally occupied by the Somali ethnic group and over 80 % of the land is earmarked for livestock production. The sparse population of approximately 7 people/km^2^ of the district is found concentrated around the water sources and also around small market centers [21, 22]. Mean annual rainfall varies between 200 and 500 mm with occasional torrential storms causing extensive flooding. Rainfall is bimodal; the long rains occurring from April and May and the short rains in October and November with occasional variation. Generally, Garissa County is hot and dry with average daily temperatures ranging from 20 to 38 °C. Lamu County is a coastal cosmopolitan area with several communities practicing diverse cultures and economic activities including small scale farming, hunting and fishing. The expansive grasslands in the region form the major attraction for nomadic pastoralists from the neighboring Garissa County, who routinely migrate into the region during drier seasons with their livestock to access pasture.

Seven sites along the LMR were sampled: Haney, Degurdei, Arbadobolo, Boni, Dondori Mlimani and Mangai (Fig. 1). The selected sites lie within three major ecological zones of Kenya. [23, 24]. Haney, Degurdei and Arbadobolo are located in the semi arid zone (Zone VI), which is characterized bydry woodland vegetation and wooded or bushed grassland. Trees in the semi arid zone are typically *Acacia* species. The vegetation in this ecological zone is patchily distributed with most plant species being well adapted to dry conditions, with the exception of herbaceous plants found growing in areas that hold water for extended durations after it rains. Boni is located within the forest ecological zone (Zone V), which is comprised of expansive indigenous woodland, with trees typically being the broad-leaved *Combretum*, alternating with patches of grassy fields. Mangai, Dondori and Mlimani areas are located within the humid to dry sub-humidzone (Zone IV), which is comprised of expansive grassland with patches of shrubby vegetation along the coastal region. The ecological zones represented in this study are important to pastoralists given that they provide pasture during different seasons and determine the livestock migration routes.

### Sampling and identification of mosquito vectors

Mosquito surveys were conducted using CO_2_-baited CDC light traps (John W. Hock Company-Model 512) during the long rains (April – June) and short rains (November – December) at each of the study sites between November 2012 and August 2014. At each of the seven sites, sampling was conducted three times during the short rains in November and long rainy season between April and May, leading to three replicates for each site per season and a total of six replicates for both seasons over the period of study. During each trapping period, ten traps were set at 1800 h and retrieved at 0600 h the following day for three consecutive sampling days at each site in both seasons. Trapped mosquitoes were anesthetized using triethylamine for ten minutes, sorted, placed into 15 ml labeled vials, and transported to the laboratory in liquid nitrogen for identification. Mosquitoes were morphologically identified to species level using taxonomic keys [25, 26].

### Statistical analysis

Data on total mosquito catches among the different ecological zones and sites were compared using a negative binomial model [27]. The mosquito captures were also compared separately across sampling sites for each of the three vector groups (primary vectors: *Ae. mcintoshi* and *Ae. ochraceus*; secondary vectors: species of the genera *Mansonia, Culex* and *Anopheles* with exception of malaria vectors; and other flood water *Aedes*: *Ae. sudanensis* Theobald and *Ae. tricholabis* (Edwards) while controlling for season (long rain = 1, short rain = 2). Mosquitoes were placed into these vector groups based on their importance/role in RVF maintenance and transmission. Risk ratios (RR) were computed for each site in comparison to Mangai, which had the highest number of mosquito catches. For ecological zones, zone 4 was taken as the reference group. Overall factor effect in the NB model was assessed using Wald test [28]. To obtain information on the rarity and commonness of vector species, we estimated the Shannon (H) and Simpson (D) diversity indices for each of the three replicate trappings during the short and long rainy seasons per site using the ‘vegan’ library [29] in R. The diversity indices were then compared across the sites and ecological zones, while controlling for season using analysis of variance (ANOVA). Species richness (that is, the number of individual species) across ecological zones, were also compared using ANOVA.We did not focus on microhabitat differences around individual traps but larger-scale ecological effects in the entire LMR, hence the reason for pooling data for each trapping period. All analyses were performed in R version 3.1.1 [30] at α = 0.05 significance level.

## Results

### Abundance of primary and secondary vectors of RVF in diverse ecological zones and sites

A total of 31,727 mosquitoes (mean = 755.5, variance = 430045.8) comprising 21 species belonging to 6 genera were captured from the 7 sampling sites (Table 1). Overall mosquito abundance varied significantly by ecological zones (Wald test = 14.8, df = 2, *P* = 0.0006). Compared to ecological zone IV, mosquitoes were significantly fewer in ecological zone VI (RR = 0.34, 95 % CI: 0.19–0.59), but not significantly different from zone V (RR = 0.54, 95 % CI: 0.26–1.26). The overall mosquito abundance also varied across sites (Wald test = 171.9, df = 6, *P* < 0.0001). The highest number of mosquitoes was trapped in Mangai in ecological zone IV (*n* = 10,740) while the lowest occurred in Haney in ecological zone VI (*n* = 282). *Aedes* was the most diverse taxon, mostly represented by the floodwater species *Ae. mcintoshi*, *Ae. ochraceus*, *Ae. tricholabis* and *Ae. sudanensis*, which were fairly well represented across ecological zones. Among the *Aedes* species, *Ae. ochraceus* and *Ae. mcintoshi* (the primary RVF vectors associated with previous outbreak) were the most abundant. While the highest number of these primary vectors of RVF occurred in zone VI (*n* = 4608), there were zone and site specific differential abundances between the two species. *Ae. ochraceus* dominated zones IV and V in Mangai, Dondori, Mlimani and Boni Forest, while *Ae. mcintoshi* was more abundant across zone VI in Haney, Degurdei and Arbadobolo (Table 2). Other vectors also important in disease circulation comprised the genus *Culex* which was mostly represented by *Culex pipiens* L with the other species in this genus occurring in much reduced numbers especially in the ecological zone VI. *Mansonia africana* Neveu-Lemaire and *Ma. uniformis* Theobald represented the genus *Mansonia* with the former occurring in higher numbers relative to *Ma. uniformis*. Although *Mansonia* species dominated the overall captures from all sites (*n* = 12,181, 38.3 %), these two vectors (*Ma. africana* and *Ma. uniformis*) were almost entirely found inecological zone IV with only 0.13 % (*n* = 16) and 0.1 % (*n* = 12) abundance in ecological zones V and VI, respectively. Anopheline species trapped during this study comprised *An. squamosus* Theobald*, An. gambiae s.l.* Giles and *An. funestus s.l.* Giles which were mainly trapped in ecological zones IV and V (Table 1).

**Table 1:** Summary of mosquito catches across the sites and ecological zones in northeastern Kenya

								Mosquito species															
Ecological Zone	Sites	*Ae. mcintoshi*	*Ae. ochraceus*	*Ae. tricholabis*	*Ae. sudanensis*	*Ma. africana*	*Ma. uniformis*	*An. squamosus*	*An. gambiae s.l*	*An. funestus s.l*	*Cx. pipiens s.l*	*Cx. poicilipes*	*Cx. univittatus*	*Cx. bitaeniorhynchus*	*Cx. tigripes*	*Cx. antenatus*	*Coquilletidia aurites*	*Ae. vitattus*	*Ae. hirsutus*	*Ae. africana*	*Ae. tarsalis*	*Ad. africana*	Total mosquitoes/site
Ecological zone IV	Mangai	190	1497	0	48	5838	593	953	785	227	408	93	56	2	21	13	9	1	0	0	5	1	10,740
(Humid to dry sub-humid)	Dondori	73	467	0	21	4994	316	315	95	28	23	10	5	1	2	3	0	0	0	0	0	0	6353
	Mlimani	210	1690	43	481	409	0	193	16	5	593	99	25	0	9	2	1	0	0	0	0	0	3776
Ecological zone V	Boni forest	495	1234	977	122	16	0	35	12	0	305	500	75	2	2	0	0	0	0	0	0	0	3775
Ecological zone VI	Degurdei	2796	86	21	148	0	0	5	3	0	999	219	141	2	17	3	0	0	0	0	0	0	4440
(Semi-arid)	Arbadobolo	923	667	491	139	0	0	0	4	0	78	2	9	0	35	11	2	0	0	0	0	0	2361
	Haney	80	56	4	22	12	0	0	0	0	40	0	0	0	0	0	0	20	22	17	6	3	282

**Table 2:** Distribution and abundance of primary vectors of RVF a cross the ecological zones and sites

Ecological zone	Sites	Primary vectors		
		*Ae. mcintoshi*	*Ae. ochraceus*	Total
Zone IV	Mangai	190	1497	1687
	Dondori	73	467	540
	Mlimani	210	1690	1900
Zone V	Boni forest	495	1234	1727
Zone VI	Haney	80	56	136
	Degurdei	2796	86	2882
	Arbadobolo	923	667	1590

Negative binomial model results comparing the abundance within each vector group across sites are presented in Table 3. The table shows a significant difference in abundance of primary vectors across the sites (Wald test = 250.4, df = 6, *P* < 0.0001) and seasons with significantly higher captures recorded during the long rains compared to the short rains (RR = 0.42, 95 % CI: 0.33–0.53, *P* < 0.0001). After controlling for season, the numbers of primary vectors caught were significantly higher in Degurdei but lower in Haney and Dondori compared to Mangai (Table 3). For the secondary vectors, the catches in all the sites were significantly lower than Mangai, after controlling for season with Haney recording the lowest abundance. The other floodwater *Aedes* group of vectors also demonstrated significant differences in the abundance of mosquitoes across the study sites, with Arbadobolo, Boni forest, Dergurdei and Mlimani recording more catches than Mangai after adjusting for season. In terms of ecologicalzones, there were no differences in abundance of primary vectors. While the secondary vectors were fewer in ecological zones V and VI compared toecological zone IV (Zone V: RR = 0.16, 95 % CI: 0.06–0.50; Zone VI: RR = 0.09, 95%CI: 0.04–0.18), there was no difference in abundance of secondary vectors between ecological zones V and VI (Wald test = 1.5, df = 1, *P* = 0.2200). The other floodwater *Aedes* were significantly less abundant in ecological zone IV (RR = 0.18, 95 % CI: 0.04–0.62) and zone VI (RR = 0.25, 95 % CI: 0.05–0.87), compared to zone V.

**Table 3:** Comparisons of catches of vectors by groups across the study sites in northeastern Kenya

Vectors group	Factors		RR (95 % CI)	*P* value
Primary vectors				
	Site	Mangai	1	
		Arbadobolo	1.07 (0.71–1.61)	0.7451
		Boni forest	1.44 (0.96–2.17)	0.0773
		Degurdei	2.10 (1.40–3.15)	0.0003
		Dondori	0.44 (0.29–0.66)	0.0001
		Haney	0.09 (0.06–0.14)	<0.0001
		Mlimani	1.10 (0.73–1.66)	0.6344
	Season	Long rain	1	
		Short rain	0.42 (0.33–0.53)	<0.0001
Secondary vectors				
	Site	Mangai	1	
		Arbadobolo	0.01 (0.01–0.01)	<0.0001
		Boni forest	0.10 (0.08–0.12)	<0.0001
		Degurdei	0.15 (0.12–0.18)	<0.0001
		Dondori	0.65 (0.55–0.78)	<0.0001
		Haney	0.01 (0.00–0.01)	<0.0001
		Mlimani	0.15 (0.12–0.18)	<0.0001
	Season	Long rain	1	
		Short rain	0.43 (0.39–0.49)	<0.0001
Other floodwater *Aedes*				
	Site	Mangai	1	
		Arbadobolo	18.9 (9.99–36.09)	<0.0001
		Boni forest	32.52 (17.25–61.91)	<0.0001
		Degurdei	3.67 (1.93–7.02)	0.0001
		Dondori	0.46 (0.21–0.99)	0.0546
		Haney	0.53 (0.25–1.11)	0.1058
		Mlimani	11.8 (6.3–22.26)	<0.0001
	Season	Long rain	1	
		Short rain	0.24 (0.17–0.34)	<0.0001

### Mosquito species diversity and richness

**Fig. 2:**
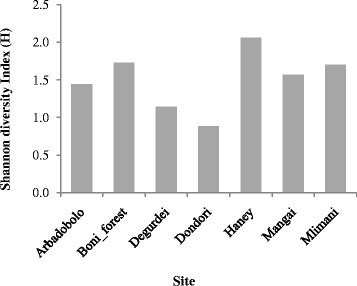
Mosquito diversity indices across the sites

Shannon diversity index showed significant differences in mosquito species diversity across the ecological zones (F = 3.33, df = 2,36, *P* = 0.0465) and sites (F = 10.82, df = 6,36, *P* < 0.0001), after controlling for season. Multiple comparisons based on Tukey’s test showed that the diversity varied significantly between zones VI and V (*P* = 0.033) but neither between zones IV and VI (*P* = 0.800) nor zones IV and V (*P* = 0.090). Mosquito diversity indices across the sites are presented in Fig. 2. Further, significantly higher mosquito diversity was observed during the long rains (H = 2.04) relative to the short rains (H = 1.85; F = 9.33, df = 1,38, *P* = 0.0041). Similar conclusions were made using Simpson diversity index.

Mosquito species richness varied significantly across the ecological zones (F = 22.98, df = 2,38, *P* < 0.0001). Ecological zone IV recorded a significantly higher number of species than ecological zone VI (*P* < 0.0001) while there was no significant difference in species richness between ecological zones IV and V. A significantly greater number of species were recorded during the long rains than short rainy season (F = 26.68, df = 1,38, *P* < 0.0001). For the sites, Mangai recorded the highest number of species (18), followed by Dondori (14) and Mlimani (14), Boni forest (12) and the rest sites with 11 species each.

## Discussion

An ecological assessment of RVF vectors is a fundamental aspect for the determination of high risk areas where emergence and circulation of RVF virus might occur. In this study, we have shown that the abundance and diversity of RVF vectors along the major nomadic livestock movement route (LMR) in northeastern Kenya vary across sites and ecological zones, which is likely to create variable points of risk for livestock exposure to the disease and subsequent human disease occurrence.

As demonstrated in this study, variation in RVF vector abundance across ecological zones indicates potential risk areas for RVF transmission and circulation. The semi arid ecological zone had a low abundance of vectors compared to other ecological zones but primary vectors of RVFv were associated with this ecological zone. The abundance of these primary vectors and other floodwater *Aedes*, especially in the semi arid zones, may be attributed to the nature of the terrain, soil types and vegetation cover, and rainfall which may influence availability of favourable vector breeding and resting grounds [19, 20]. Our study also shows that more of the vectors were trapped during long rains than short rains across all the three ecological zones which may aid the amplification of RVF virus during epidemics. This finding is in agreement with other studies which also pointed out that during periods of rainfall, mass emergence of mosquitoes may occur in their preferred breeding grounds and lead to epizootics of RVF [31]. Differential distribution patterns of RVF vectors may play an important role in understanding the epidemiology of RVFv. However, it is still unclear what causes the differential abundance among the vectors and how this may impact on RVF risk in sites along the LMR. The observed pattern suggests that large scale differences in environmental conditions possibly influence the choice of sites colonized by these vectors, and differences in the abundance of each species may drive RVF virus transmission separately at different sites, influencing levels of virus activity in different sites along the LMR. *Aedes ochraceus* has only recently been implicated as a primary vector of RVFv in northern Kenya, having been involved in circulation of the virus during the 2006/7 outbreak [20]. The high abundance of *Ae. ochraceus* in ecological zone IV and V suggests the potential suitability of such environments for this species, meaning that *Ae. ochraceus* may drive the transmission of arbovirus in these ecological zones. Recent genetic analysis has also documented population expansion of this species in Kenya, with potential for greater epidemiological importance in future RVF outbreaks [32]. Similarly *Ae. mcintoshi* could also play an important role in the semi-arid ecological zone where it was the most abundant primary vector.

Our data showed an overall low occurrence of *Culex* mosquitoes, especially species known to play secondary roles in the transmission of RVFv such as *Cx. poicilipes* Theobald and *Cx. univittatus* Theobald*.* However, it was notable that there was variation in abundance of secondary vectors across the ecological zones. Vector populations involved in the circulation of RVFv are known to show a succession pattern with the emergence first of floodwater *Aedes* (primary vectors) whose populations are gradually replaced by those of secondary vectors comprising members of the genera *Culex*, *Mansonia,* and *Anopheles* [13]. The low occurrence of secondary vectors along the LMR in our study concurs with earlier studies conducted in parts of the Ijara region of northeastern Kenya [19, 20]. However, the widespread distribution of species such as *Cx. pipiens s.l* suggests its high level of adaptability to various ecological conditions in this region [33]. Due to this, *Cx. pipiens s.l* may be amongst the most important secondary vectors for amplification of the virus during epidemics in the northeastern region of Kenya, as was the case during epidemics in Egypt [34].

The clear preference of *Mansonia* species, which are secondary vectors of RVF [2], to sites within the humid to dry sub-humid ecological zone may be related to their biology. These vectors were trapped atsites associated with marshy environments, which are characteristic of ecological zone IV in the coastal regions of Kenya. Even though their distribution was not wide spread, *Ma. africana* and *Ma. uniformis* could also play important roles as amplifiers of RVFv in the coastal region of Kenya when the virus is introduced by livestock moving from potential virus circulation zones [35].

Our study also revealed that the mosquito assemblages along the LMR had high species diversity and richness. As expected, the species diversity and richness of the RVF vectors was higher during the long rains relative to the short rainy season across the ecological zones. This was likely due to an increased number of vector breeding habitats during long rains, which may have favoured the emergence of many vectors. This finding corroborates results of previous studies conducted during RVF outbreaks in the same region, which highlighted the potential role of prolonged rainfall and mass emergence of mosquitoes as one of the risk factors leading to the severe RVF epidemic in 2007 [17]. Higher diversity in ecological zone V compared to VI observed during this study could be attributed to the variation in climatic and environmental conditions between these ecological zones, which could potentially influence the adaptation of mosquito species populations in such areas [36, 37]. Forest ecological zone V may, for instance, create humid conditions that improve survival of vector species in comparison with the extreme dry and hot conditions in the semi arid ecological zone VI. Other factors could also include differences in anthropogenic activities, including opening up water points for livestock in the forests,which could potentially influence mosquito breeding patterns between the ecological zones and promote mosquito diversity [38]. It is however, important to note that we found fewer mosquito species than in earlier studies conducted in Ijara areas of northeastern Kenya [19]. Factors that may contribute to this difference are the choice of sampling sites along LMR, frequency of sampling employed, method of sampling and duration of our study, which spanned a period of only two years. As such, a long-term, longitudinal study with more spatial replication will be required to unravel potential changes in the mosquito fauna across different seasons and sites in this region.

## Conclusion

In conclusion, our study showed the widespread occurrence of both primary and secondary vectors of RVFv in varying abundance and diversity across sites and ecological zones on the livestock movement routes used by nomadic pastoralists in northeastern Kenya. This may be important for understanding the epidemiology of RVF together with other mosquito-borne diseases in Northern Kenya. This pattern is likely to create variable risk areas of the disease with regards to infection of susceptible livestock. Mapping of these sites can be provided to the authorities for the purpose of implementing a focused RVF vector control and as a guide to formulating strategic animal vaccination plans for RVF prevention.
